# Multivoxel pattern analysis reveals dissociations between subjective fear and its physiological correlates

**DOI:** 10.1038/s41380-019-0520-3

**Published:** 2019-10-29

**Authors:** Vincent Taschereau-Dumouchel, Mitsuo Kawato, Hakwan Lau

**Affiliations:** 1 Department of Decoded Neurofeedback, ATR Computational Neuroscience Laboratories, Kyoto, 619-0288 Japan; 2 Department of Psychology, UCLA, Los Angeles, CA 90095 USA; 3 RIKEN Center for Advanced Intelligence Project, ATR Institute International, Kyoto, Japan; 4 Brain Research Institute, UCLA, Los Angeles, CA 90095 USA; 5 Department of Psychology, University of Hong Kong, Pokfulam Road, Pok Fu Lam, Hong Kong; 6 State Key Laboratory of Brain and Cognitive Sciences, University of Hong Kong, Kowloon Tong, Hong Kong

**Keywords:** Neuroscience, Psychiatric disorders

## Abstract

In studies of anxiety and other affective disorders, objectively measured physiological responses have commonly been used as a proxy for measuring subjective experiences associated with pathology. However, this commonly adopted “biosignal” approach has recently been called into question on the grounds that subjective experiences and objective physiological responses may dissociate. We performed machine-learning-based analyses on functional magnetic resonance imaging (fMRI) data to assess this issue in the case of fear. Although subjective fear and objective physiological responses were correlated in general, the respective whole-brain multivoxel decoders for the two measures were different. Some key brain regions such as the amygdala and insula appear to be primarily involved in the prediction of physiological reactivity, whereas some regions previously associated with metacognition and conscious perception, including some areas in the prefrontal cortex, appear to be primarily predictive of the subjective experience of fear. The present findings are in support of the recent call for caution in assuming a one-to-one mapping between subjective sufferings and their putative biosignals, despite the clear advantages in the latter’s being objectively and continuously measurable in physiological terms.

## Introduction

Physiological markers have been used as proxies for psychological states in multiple mental health domains. However, evidence accumulated over the years called into question the relationship between some subjective mental states and their proposed physiological markers. For example, in the case of pain, it is well established that subjective nociceptive experiences can occur without any obvious peripheral physiological manifestations [1]. As a result, the self-reported subjective experience remains to this day the gold standard in pain assessment [2].

Currently, a similar debate is taking place concerning fear and anxiety [3–5]. In that literature, physiological reactivity to threat has been considered a reliable objective proxy for the subjective experience of fear [6]. The reliance on such physiological measures proved to be quite successful and they are now included in numerous studies on fear and anxiety [7]. Specifically, the neural network involved in physiological reactivity is currently one of the primary neurobiological targets for the pharmacological treatment of anxiety disorders [8].

However, some authors suggest that physiological reactivity (as commonly indexed by skin conductance and amygdala reactivity) might represent automatic, defensive responses that are not necessarily conscious [3, 4, 9]. On this view, studying these physiological defensive responses may not cover all the relevant mechanisms involved in the subjective suffering that is central to fear and anxiety disorders. Accordingly, it is argued that an overemphasis on objective physiological biosignals might slow down the development of new therapeutic options [4]. This position remains controversial as others have pointed out that multiple lines of evidence actually indicate a high correlation between subjective fear reports and physiological responses, notably in the amygdala [5]. Here we attempt to bring in evidence to arbitrate this debate using human functional neuroimaging.

**Fig. 1:**
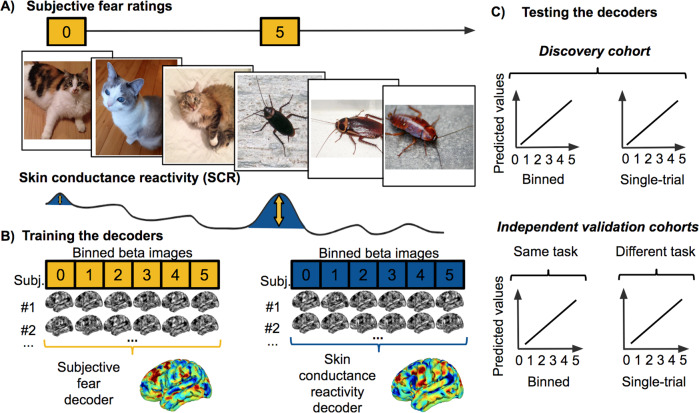
Experimental design and decoding procedure. **a** We recorded functional brain activity and electrodermal activity during the presentation of images depicting 30 animal categories and 10 man-made objects. The skin conductance reactivity was established during the fMRI session using standard analytical procedures (see Methods). The subjective fear ratings were established before the fMRI procedure without presenting any fearful stimuli. This approach was similar to the typical clinical procedures used for the assessment of fear. **b** The images were presented in chunks of 2, 3, 4, or 6 images of the same category. The fMRI analyses modeled the first images of each chunk, because they could be attributed both a subjective fear rating and a level of skin conductance reactivity (see Methods). The estimated brain responses were binned (i.e., averaged) as a function of their categorical fear ratings (left) or skin conductance reactivity (right). The binned beta images of the discovery cohort were used to train the decoders. The unthresholded weight maps of the whole-brain decoders are displayed. **c** The performances of the decoders were tested in the discovery cohort (both on binned and single-trial data) as well as in independent validation cohorts not included in the training of the decoder. This procedure allowed us to estimate the generalization of the decoders to new datasets. The first independent cohort included new participants (*N* = 12) performing the same task as the one performed by the discovery cohort. The second independent cohort (*N* = 17) performed a different experimental task where pictures of feared animals were also presented (see Supplementary Methods and Results)

Specifically, our goal is to study whether the brain representation of subjective fear ratings dissociates from the representation of objective physiological reactivity (i.e., skin conductance response to feared images). To do so, we focused on naturally occurring instead of conditioned fears. One advantage is that these representations are likely to reflect more closely the brain mechanisms involved in anxiety disorders such as naturally occurring phobia. We constructed a functional magnetic resonance imaging (fMRI) experiment to present as many as 3600 images of the most commonly feared animals, some neutral animals, as well as some man-made objects as controls (see Fig. 1a).

We used a machine-learning approach [10–12] to train multivoxel brain decoders to predict either objective physiological reactivity or subjective fear reports. To do so, we leveraged whole-brain data to determine the patterns of voxel activities that are the most predictive of each outcome (i.e., levels of fear and levels of skin conductance reactivity). We then tested the generalization of these decoders using two independent validation datasets (*N* = 12 and *N* = 17) (see Fig. 1c) and the brain data of patients diagnosed with specific phobias (*N* = 3). Furthermore, we aimed at determining whether some brain regions are preferentially involved in the prediction of either the subjective or physiological measures. As such, we established where in the brain it was possible to predict one outcome with a better accuracy than the other. This was achieved by comparing the predictions of both decoders within brain regions.

To anticipate, we found that the representations of subjective fear and skin conductance reactivity present some overlap but also some differences in the brain. Specifically, regions previously associated with defensive responses (such as the amygdala) present a preference in the prediction of defensive responses, whereas some higher-order frontal regions, previously associated with conscious perception and metacognition, appear primarily involved in the prediction of the subjective fear reports.

## Methods

### Participants

The discovery cohort included 31 participants (15 females; mean age = 23.29 years; SD = 4.21). Participants were included if they reported, on a six-point Likert scale, “high” or “very high” fear of at least one animal included in the experiment (see “Stimuli and task” for a detailed list). Skin conductance reactivity was not acquired for four participants and technical issues prevented from recording the skin conductance of two participants. As a result, the data of 25 participants were available to train the skin conductance reactivity decoder. The first independent validation cohort included 12 participants (2 females; mean age = 25.75 years; SD = 3.98) who performed the same fMRI procedure (i.e., same task in Fig. 1c). Skin conductance reactivity was acquired for eight of them. The second independent validation cohort task comprised 17 participants from the discovery cohort (5 females; mean age = 21.92 years; SD = 1.54) that performed a different experimental task (i.e., different task in Fig. 1c) (see Supplementary Methods). Skin conductance reactivity was recorded for all participants.

The sampling procedure was based on convenience. The sample sizes were set according to the number of participants included in previous studies [13, 14]. No acquired data were excluded from the analysis. No participants dropped out or declined participation. Participants were not allocated in experimental groups. All participants provided written informed consent and the study was approved by the Institutional Review Board of Advanced Telecommunications Research Institute International (ATR), Japan.

### Stimuli and task

The experimental procedure has been described in detail elsewhere [13] and is summarized in Fig. 1a. Briefly, participants underwent a 1 h fMRI session where they were presented with images of the most commonly feared animals (e.g., snake, spider, cockroach, bee, bat, mouse, dog, cat, shark, etc.) as well as pictures of other animals and objects. We chose to present 90 different images per category and to include 30 animal categories and 10 object categories (for a total of 3600 different images). The 30 different animal categories included reptiles (snake, turtle, and gecko), amphibians (frog), insects (cockroach, beetle, ant, spider, grasshopper, caterpillar, bee, butterfly, and fly), birds (robin, peacock, and chicken), annelids (earthworm), mammals (mouse, guinea pig, bat, dog, sheep, cat, rabbit, horse, and giraffe), and aquatic animals (shark, whale, common fish, and dolphin). The database also included ten categories of human-made objects (airplane, car, bicycle, scissors, hammer, key, guitar, cellphone, umbrella, and chair). The images presented a full frontal view of the object or animal and no other recognizable object was clearly identifiable in the background. Images were cropped so that they would frame the object. The final images were 533 × 533 pixels and covered 13.33° of visual angles during the procedure. The average contrast and luminance of images were not different between categories [13]. The data of the human-made objects were not analyzed. Trials were organized in six runs of 600 trials interleaved with short breaks. The sequence of presentation was pseudo-randomized and fixed across participants.

### MRI parameters

Participants were scanned in two 3T MRI scanners (Prisma Siemens and Verio Siemens) with a head coil at the ATR Brain Activation Imaging Center. During the experiments, we obtained 33 contiguous slices (Repetition time (TR) = 2000 ms, Echo time (TE) = 30 ms, voxel size = 3 × 3 × 3.5 mm^3^, field-of-view = 192 × 192 mm, matrix size = 64 × 64, slice thickness = 3.5 mm, 0 mm slice gap, flip angle = 80°) oriented parallel to the AC-PC plane, which covered the entire brain. We also obtained T1-weighted MR images (MP-RAGE; 256 slices, TR = 2250 ms, TE = 3.06 ms, voxel size = 1 × 1 × 1 mm^3^, field-of-view = 256 × 256 mm, matrix size = 256 × 256, slice thickness = 1 mm, 0 mm slice gap, TI = 900 ms, flip angle = 9°).

### Recording of electrodermal activity

Skin conductance reactivity was determined during the fMRI sessions using BrainAmp Ag/AgCl sintered MR electrodes (Brain Products). The electrodes were disposed on the distal phalanges of the index and middle fingers of the left hand. Skin conductance reactivity was determined in response to the first image of each chunk of images of a given category. Following previous methodologies [13], we determined the maximum amplitude in a time window of 1–5 s following the image onset and removed from this value the baseline activity in a 2 s window before the image onset. Responses smaller than 0.2 microsiemens (μS) were recoded as 0 (see Supplementary Methods). Responses were square-root transformed to correct for the skewness of the distribution [15]. This standard analytical procedure allowed for our results to be readily put in correspondence with previous findings. However, this approach presents the disadvantage of allowing for the peak of skin conductance reactivity of one trial to occur within the time window of the following trial. We quantified that this scenario happened in 2.62% of all trials. This did not prevent us from developing a sensitive and accurate decoder of the skin conductance reactivity (see Figs. 3, 4, and Supplementary Figs. S1 and S2). However, this represents a source of noise that could be avoided in future experiments by using longer presentation chunks.

### Comparing subjective fear ratings and skin conductance reactivity

**Fig. 2:**
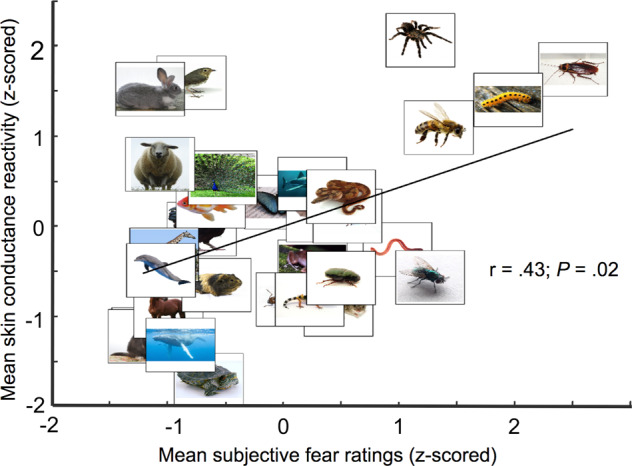
Skin conductance reactivity is correlated with subjective fear ratings. Within each category, subjective fear ratings and mean skin conductance reactivity were averaged at the group level and standardized (see Methods). As expected, skin conductance reactivity was correlated with subjective fear ratings (*r*(28) = 0.43; *P* = 0.02; 95% CI: 0.08–0.69; *R*^2^ = 0.19; two-sided)

To determine the correlation between subjective fear reports and skin conductance reactivity, we first established, for each participant, an average level of skin conductance reactivity for each animal category. Since the first trial of each run (i.e., trials were organized in 6 runs of 600 trials) was typically associated with greater skin conductance reactivity, we removed these six trials as they did not represent a typical reactivity to the image category per se. This removed 6 out of the 720 trials. The remaining trials were winsorized (5th and 95th percentile), averaged within category and standardized. We then established group-level mean value for each category by averaging across participants. This was achieved both for the subjective fear ratings and for the skin conductance reactivity. The mean categorical values were then standardized at the group level. These standardized values were correlated to determine the association between subjective fear ratings and skin conductance reactivity at the group level. The results are presented in Fig. 2.

### Preprocessing of fMRI data

The fMRI images captured during the experiment were realigned to the first fMRI image, coregistered, and motion-corrected (using six motion parameters) in SPM 12 (Statistical Parametric Mapping; www.fil.ion.ucl.ac.uk/spm) [16]. Functions of pyMVPA (www.pymvpa.org) [17, 18] implemented in the Neurodebian environment [19] were used to remove the linear trend and to deconvolve the signals using the least-square separate approach [20, 21]. This method allowed to iteratively fit a general linear model to estimate the brain response to the first presentation of each chunk of images. Each general linear model includes one parameter modeling the current trial and two parameters modeling all other trials in the design. Via this method, we were able to obtain one parameter estimate (i.e., a beta image) for each individual trial of our rapid-event-related design (720 beta images for each participant). Data were also normalized to the Montreal Neurological Institute (MNI) space and smoothed (full-width at half maximum = [8,8,8]) using SPM 12.

### Whole-brain decoders

To train whole-brain decoders in the discovery cohort, we created two datasets by binning (i.e., averaging) together within-subject beta images either as a function of individual fear ratings (0 = “No Fear” to 5 = “Very High Fear”) or as a function of skin conductance reactivity (according to individual quintiles of the reactivity) (see Fig. 1). This procedure allowed both to remove the effect of outliers and to capture the within-subject variability of each measure. To train the subjective fear decoders, the binned beta images were created by binning together, within-participant, the trials corresponding to the same level of fear (0 = “No fear” to 5 = “Very high fear”). A similar procedure was used to create the binned beta images to train the skin conductance reactivity decoder. We aimed at creating six binned images per participant to reflect the different skin conductance reactivity levels. However, because of the skewness of the distribution, splitting the data according to even quintiles would result in an over-representation of the trials with very small reactivity (i.e., most of the trials are below 0.2 μS). As such, trials below 0.2 μS were considered to be part of the binned beta image of level 0. The remaining trials were grouped into quintiles (computed individually) and the binned beta images 1–5 were obtained by averaging the corresponding images together. The number of trials in each bin was used to set the number of trials randomly selected to constitute the binned image of level 0. Binned beta images were mean centered within subject.

**Fig. 3:**
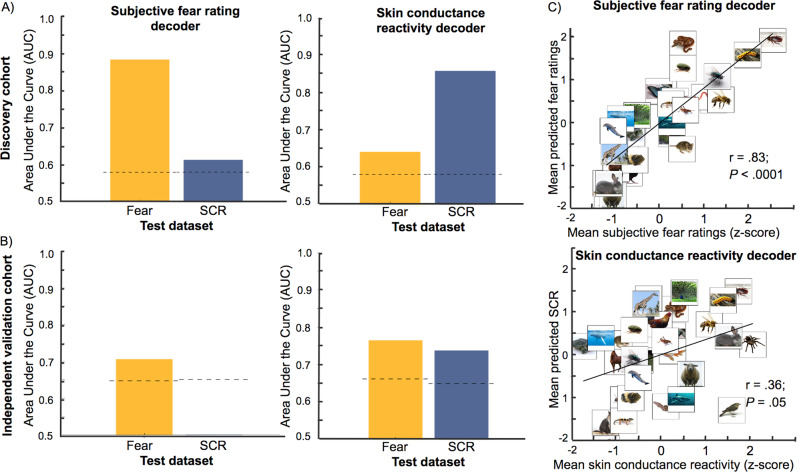
Whole-brain decoders of subjective fear and skin conductance reactivity. **a** Both whole-brain decoders presented a good sensitivity when tested on the dataset they were trained to predict (e.g., subjective fear decoder predicting the fear dataset). The cross-decoding procedure (e.g., predicting skin conductance reactivity using the subjective fear decoder) also revealed that both decoders can generalize to some extent to the other dataset. Dashed lines represent the critical value (*p* = 0.05) determined using a permutation test. **b** Both whole-brain decoders also generalized to new data as evidenced by their good capacity to predict the independent validation cohort. The cross-decoding procedure indicated that the skin conductance decoder could also predict accurately the subjective fear rating dataset (right panel). This was not observed for the subjective fear decoder (left panel). **c** The whole-brain decoders were also tested on the categorical beta images of each participant (the prediction of the subjective fear decoder and subjective fear ratings: *r*(28) = 0.82; *P* < 0.0001; 95% CI: 0.65–0.91; *R*^2^ = 0.67; two-sided; the predictions of the skin conductance decoder and the skin conductance reactivity of the categories: *r*(28) = 0.36; *P* = 0.05; 95% CI: −0.006–0.63; *R*^2^ = 0.13; two-sided)

**Fig. 4:**
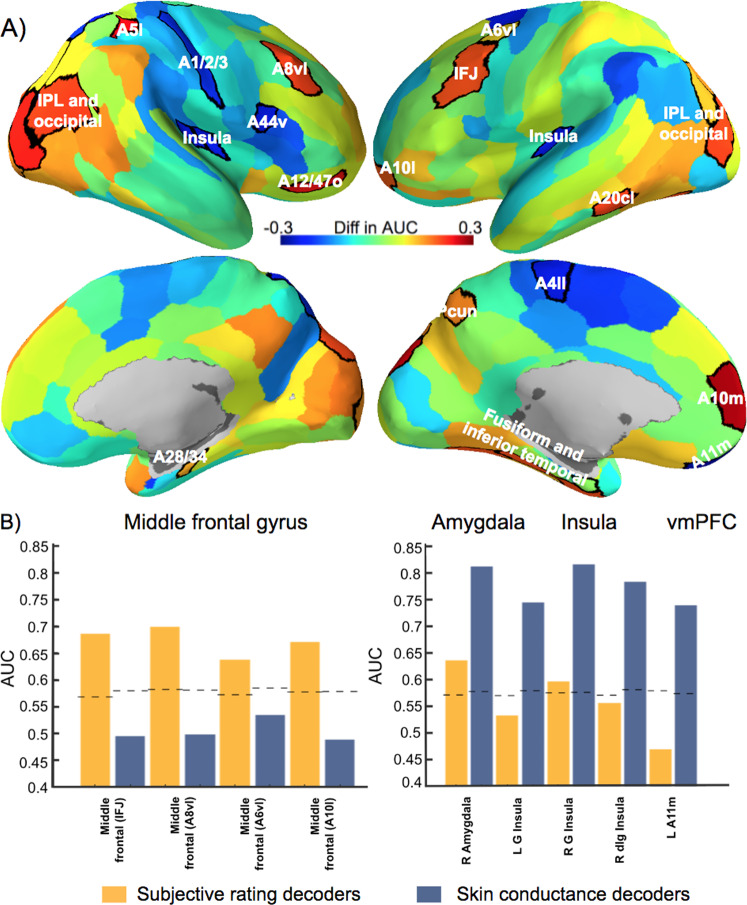
Brain regions presenting a significant difference in the prediction of the subjective ratings and skin conductance reactivity. **a** A positive difference in the area under the curve indicates a better prediction of the subjective ratings (red–orange regions) whereas a negative difference indicates a better prediction of skin conductance reactivity (blue regions). The significant regions (*p* < 0.05; FDR-corrected) are surrounded by black borders and are listed in Table 1. Brain images were generated using pySurfer (https://pysurfer.github.io/). **b** Significant regions of the middle frontal gyrus, amygdala, insula, and ventral medial prefrontal cortex (vmPFC). Dashed lines represent the critical value (*p* = 0.05) determined using a permutation test

In a cross-validation procedure, we trained a support vector regression decoder on the data of N-1 participants and tested the accuracy of the decoder to predict the left-out participant (i.e., leave-one-subject-out cross-validation approach) (implemented in Matlab [https://www.mathworks.com/products/matlab.html] using the CanlabCore toolbox [https://github.com/canlab/CanlabCore] and the Spider machine-learning library [http://people.kyb.tuebingen.mpg.de/spider/main.html]). This procedure was achieved iteratively to obtain predicted values for all participants. We established both the sensitivity (e.g., can we predict accurately the subjective ratings of fear?) and the specificity (e.g., can we predict the subjective ratings with the skin conductance reactivity decoder?) of each whole-brain decoders. The sensitivity was established using the area under the receiver operating characteristic curve (AUC) of the predicted values. To determine the statistical significance of the AUC, we conducted a permutation test by randomly permuting (1000 times) the labels of the beta images in the datasets. Applying the decoders to this permuted data allowed to obtain a distribution of AUCs under the null hypothesis. This was achieved to obtain a critical value for significance at *p* = 0.05 (dashed lines in Figs. 3a, 4b). The specificity was also determined by testing each decoder using the dataset of the other outcome (e.g., testing the subjective fear rating decoder using the skin conductance dataset). This process, which we call “cross-decoding,” can reveal similarities between brain representations if the results reveal above-chance performances. We also determined the performance of the decoders trained with binned beta images in the prediction of single-trial (i.e., unaveraged) beta images. This was achieved also using a leave-one-subject-out cross-validation procedure (i.e., training with binned beta images and testing with single-trial data of the left-out participant) (see Supplementary Methods and Supplementary Fig. S1).

As participants presented various levels of fear and skin conductance response to each category, the prediction of categorical beta images (e.g., averaged beta images of snakes) by each decoder should also follow the subjective ratings and skin conductance reactivity. To test this hypothesis, we computed mean beta images for each animal category and submitted these beta images to the decoders. We generated binned categorical images by removing the first trial of each block from the binned beta images (see above Comparing subjective ratings and skin conductance reactivity). This resulted in removing 6 trials out of the 720 beta images. We submitted the average categorical images to the whole-brain decoders. The predicted values were winsorized (5th and 95th percentile) and standardized within participant. At the group level, these values were averaged, standardized, and then correlated with the mean subjective fear ratings and skin conductance reactivity (see Fig. 3c).

### Testing the generalization of whole-brain decoders

Although leave-one-subject-out cross-validation is a common practice in machine learning, this approach may not reflect the true generalization capability of the decoders [22]. As such, we estimated the generalization of the decoders using two independent validation datasets as well as patients diagnosed with specific phobia.

The first dataset included a group of participants (*N* = 12) that went through the same fMRI procedure (i.e., same task) but were not included in the training of the decoder [23].

The second independent validation dataset was composed of a subsample of participants from the discovery cohort (*N* = 17) that took part in a new fMRI procedure (i.e., different task) (see Supplementary Methods). In this experiment, participants were asked to assess online their subjective fear of images of feared and non-feared animals. Their skin conductance reactivity was recorded during this fMRI procedure. We processed single-trial beta images from this experiment and submitted these images to the whole-brain decoders (Supplementary Fig. S2 and Methods).

Another important question pertains to the generalization of the brain decoders to a clinical population. To provide some information regarding this generalization, we included in the study three patients (*N* = 3) diagnosed with specific phobia of one of the 30 animals (see Supplementary Methods and Supplementary Fig. S3).

### Within-region decoders

We also aimed to determine the brain regions differentially involved in the prediction of subjective fear ratings and skin conductance reactivity. Thus, we used the same leave-one-subject-out cross-validation but within predefined brain regions. For this purpose, we used a parcellation of the cortex based on functional connectivity [24]. We selected the 210 cortical regions of this brain atlas, as well as the amygdala and hippocampus, for a total of 214 regions. We iteratively trained decoders within each of the selected regions to predict either outcome. This procedure provided us with a correlation coefficient between the predicted and real values for each decoder, within each region. This allowed for a direct comparison of the correlation coefficients between decoders using Fisher’s method [25]. As such, this procedure was used to determine where in the brain one decoder presented a better performance than the other (e.g., a better prediction of the subjective ratings than the skin conductance reactivity). These statistical comparisons were corrected to account for multiple comparisons. The false discovery rate of this series of dependent tests was controlled using the method described by Yekutieli and Benjamini [26], and was implemented using the Matlab toolbox fdr_bh (http://kutaslab.ucsd.edu/matlabmk_fn_docs/matlabmk/fdr_bh.html). To facilitate the interpretation, we plot in Fig. 4a the difference in the AUCs of both decoders within each region.

We also determined the brain regions that could reliably represent both outcomes. Using the within-region decoding procedure, we conducted an exploratory conjunction analysis to determine the brain regions that were involved in the correct prediction of both subjective fear ratings and skin conductance reactivity (see Supplementary Methods, Results, and Supplementary Table S1).

## Results

As expected based on previous literature, subjective fear ratings and skin conductance reactivity were correlated (*r*(28) = 0.43; *P* = 0.02; 95% confidence interval (CI): 0.08–0.69; *R*^2^ = 0.19; two-sided) (see Fig. 2 and Methods). The data met the assumptions of the test. No outlier was observed, the data were normally distributed, and the data presented no heteroscedasticity. Both outcomes also presented some level of variability. At the group level, 59% of trials were associated with a certain level of fear (4.6% very high fear, 11.0% high fear, 12.8% moderate fear, 15.9% low fear, and 14.7% very low fear), whereas 41% were associated with no fear. Regarding skin conductance reactivity, 28.21% of trials were considered to present a certain level of reactivity (>.2 μS), whereas 71.79% did not (see Supplementary Methods).

### Whole-brain decoders

Figure 3a shows the discrimination accuracy of the whole-brain decoders of subjective fear ratings (Fig. 3a, left panel) and skin conductance reactivity (Fig. 3a, right panel). Both decoders present a high level of sensitivity in the prediction of binned images (AUCs ~ 0.85) as well as a good sensitivity (AUCs ~.62) in the prediction of single-trial images of the discovery cohort (see Fig. S1 and Supplementary Methods and Results). Both decoders also showed some cross-decoding capacity as indicated by the above-chance classification of the binned images (dashed lines in Fig. 3 correspond to *p* = 0.05 determined with a permutation test).

The results also indicated that whole-brain decoders presented significant predictions of the categorical beta images. More precisely, at the group level, the predictions of the subjective fear decoder were correlated with the subjective fear ratings (*r*(28) = 0.82; *P* < 0.0001; 95% CI: 0.65–0.91; *R*^2^ = 0.67; two-sided) (see Fig. 3c, top panel) and the predictions of the skin conductance decoder were correlated with the average skin conductance reactivity of the categories (*r*(28) = 0.36; *P* = 0.05; 95% CI: −0.006–0.63; *R*^2^ = 0.13; two-sided) (see Fig. 3c, bottom panel). The data met the assumptions of the tests. No outlier was observed, the data were normally distributed, and the data presented no heteroscedasticity.

### Testing the generalization of whole-brain decoders

Figure 3b shows the discrimination accuracy of the subjective fear (Fig. 3a, left panel) and skin conductance reactivity decoders (Fig. 3a, right panel) in the prediction of the first independent validation cohort (*N* = 12) (e.g., new participants performing the same fMRI task). Both decoders presented sensitivity (AUCs ~ 0.70) in the prediction of the outcome they were trained to predict. There was also an above-chance classification of the subjective fear dataset using the skin conductance decoder (dashed lines in Fig. 3 correspond to *p* = 0.05). Regarding the second independent validation dataset (*N* = 17) (i.e., participants performing a different task), both decoders presented weak but statistically significant predictions (see Fig. S2 and Supplementary Methods and Results). Furthermore, the decoders presented a similar level of accuracy as the rest of the discovery cohort when tested using the data of patients diagnosed with specific phobia (see Fig. S3 and Supplementary Methods and Results).

Taken together, these results indicate that it is possible to develop sensitive whole-brain decoders of subjective fear and skin conductance reactivity. Importantly, our results suggest that both decoders can generalize, to some extent, to two independent validation cohorts as well as to patients diagnosed with specific phobia. Furthermore, the predictions of the decoders appear to correspond to the individual variability in the dataset as assessed by the prediction of the categorical beta images of each animal category. Although these decoders present some similarities, they also appear to be independent of one another as indicated by the results of the cross-decoding procedure.

### Within-region decoders

Figure 4 and Table 1 indicate in which brain regions the predictions of the decoders were statistically different. Interestingly, the significant regions of the middle frontal gyrus (inferior frontal junction, A8vl, A6vl, and A10l) all involved a better prediction of the subjective fear ratings than the skin conductance reactivity (see Fig. 4b, left panel). Other regions presenting such a preference for the prediction of the subjective ratings include the medial superior frontal gyrus, the lateral orbitofrontal gyrus, the inferior temporal gyrus, the fusiform gyrus, the parahippocampal gyrus, the superior parietal lobule, the inferior parietal lobule, the precuneus, and the occipital lobe (see Table 1). Furthermore, other regions such as the amygdala, the insula, and the ventral medial prefrontal cortex appear to be primarily associated with the skin conductance response, while being marginally involved in the prediction of the subjective fear ratings (see Fig. 4b, right panel). Other regions presenting such a preference are the lateral inferior frontal gyrus, the superior parietal lobule, the paracentral lobule, and the postcentral gyrus. As expected from this series of correlational analyses, some tests did not fulfill all the Pearson’s correlation assumptions. However, when robust correlations are conducted (skipped correlations [27–30]), the interpretation remains qualitatively the same. These results suggest that the subjective experience of fear might involve brain processes partly distinct from those involved in the production of the skin conductance response.

**Table 1:** Regions presenting a significant difference in the prediction of subjective ratings and skin conductance responses

*Z*	*P*	Gyrus	Region	Laterality	MNI coordinates [*X*,*Y*,*Z*]
Fear > SCR					
4.063	0.000048	Superior frontal	Medial area A10m	L	−8, 56, 15
3.960	0.000075	Middle frontal	Inferior frontal junction (IFJ)	L	−42, 13, 36
3.832	0.000127		A8vl, ventrolateral area 8	R	42, 27, 39
3.457	0.000546		A6vl, ventrolateral area 6	L	−32, 4, 55
3.836	0.000125		A10l, lateral area 10	L	−26, 60, −6
4.505	0.000007	Orbital	A12/47o, orbital area 12/47	R	40, 39, −14
3.314	0.000921	Inferior temporal	A37elv, extreme latero-ventral area 37	L	−51, −57, −15
3.718	0.000201		A20cl, caudolateral of area 20	L	−59, −42, −16
3.942	0.000081	Fusiform	A20rv, rostroventral area 20	L	−33, −16, −32
3.427	0.000610		A37mv, medioventral area 37	L	−31, −64, −14
3.558	0.000373	Parahippocampal	A35/36c, caudal area 35/36	R	26, −23, −27
3.582	0.000341	Superior parietal lobule	A5l, lateral area 5	R	35, −42, 54
3.688	0.000226	Inferior parietal lobule	A39c, caudal area 39(PGp)	L	−34, −80, 29
3.845	0.000120		A39c, caudal area 39(PGp)	R	45, −71, 20
3.991	0.000065	Precuneus	A7m, medial area 7(PEp)	L	−5, −63, 51
4.701	0.000002	Occipital lobe	mOccG, middle occipital gyrus	L	−31, −89, 11
4.48	0.000007		mOccG, middle occipital gyrus	R	34, −86, 11
3.940	0.000081		OPC, occipital polar cortex	R	22, −97, 4
4.795	0.000002		msOccG, medial superior occipital gyrus	L	−11, −88, 31
3.488	0.000487		msOccG, medial superior occipital gyrus	R	16, −85, 34
3.615	0.000301		lsOccG, lateral superior occipital gyrus	L	−22, −77, 36
3.36	0.000755		lsOccG, lateral superior occipital gyrus	R	29, −75, 36
SCR > Fear					
−3.355	0.000794	Amygdala	Medial and lateral amygdala	R	−23, −3, −20
−3.844	0.000121	Inferior frontal	A44v, ventral area 44	R	54, 14, 11
−4.068	0.000047	Orbital	A11m, medial area 11	L	−6, 52, −19
−3.864	0.000112	Paracentral lobule	A4ll, area 4, (lower limb region)	L	−4, −23, 61
−3.860	0.000113	Superior parietal lobule	A7r, rostral area 7	R	19, −57, 65
−3.551	0.000384		A7c, caudal area 7	R	19, −69, 54
−3.446	0.000569	Postcentral	A1/2/3ulhf, area 1/2/3(upper limb, head and face region)	R	50, −14, 44
−3.441	0.000579	Insular	G, hypergranular insula	L	−36, −20, 10
−4.318	0.000016			R	37, −18, 8
−4.598	0.000004		dIg, dorsal granular insula	R	39, −7, 8

Following Fisher’s method [25], the *Z*-value can be used to compare directly the correlations between the predicted values of each decoder and the real values

Furthermore, an exploratory conjunction analysis indicated that some brain regions could present a significant prediction of both outcomes. These regions notably include part of the inferior frontal gyrus, insula, precuneus, hippocampus, and fusiform gyrus (see Supplementary Results and Supplementary Table S1).

## Discussion

Our results are in line with multiple previous findings, indicating a positive relationship between the subjective fear ratings and autonomic responses [31–34]. However, here we also showed that the brain regions involved in the accurate prediction of these two measures are possibly distinct. For instance, brain regions such as the amygdala, insula, and ventromedial prefrontal cortex appeared mostly involved in the prediction of physiological reactivity (Fig. 4b, right panel), whereas regions of the middle frontal gyrus, dorsomedial prefrontal cortex, and lateral orbital cortex were more closely related to the subjective reports of fear (Fig. 4b, left panel). This suggests that some caution may be warranted in the use of physiological reactivity as the sole source of information to infer the subjective suffering associated with fear and anxiety disorders.

Our results raise the question of the relation between physiological reactivity and subjective fear in the brain. To what extent are their representations independent? Is the subjective fear rating a late-stage readout of the physiological reactivity? Similar questions have been previously discussed in the consciousness literature [35, 36]. For instance, Maniscalco and Lau [36] tested multiple models formalizing the potential relations between the sensory signal and subjective judgment. Their results suggest that a hierarchical model in which subjective experience depends on late-stage readout best accounted for the data. This is notably in line with higher-order [3, 35, 37] and constructivist theories [38, 39] of emotions, suggesting that first-order representations (possibly reflected by the physiological reactivity) may need to be attended or meta-represented downstream for subjective experiences to occur. This is also in accord with a recent review of the literature [40], indicating that a meta-representation of the lower-level affective processes might be implemented by the middle frontal gyrus and other areas in the lateral prefrontal cortex.

Further evidence for a hierarchical model comes from results, indicating that low-level affective processes do not seem necessary to generate a conscious experience of fear. For instance, patients presenting bilateral lesions of the amygdala have been reported to be capable of experiencing fear in some specific situations [41, 42]. Although there is still some debate regarding the possible mechanisms leading to these subjective experiences, the overall evidence at least does not seem incompatible with higher-order models.

A higher-order perspective is in line with previous results [12, 41–45] and ours, but models of emotions are still being debated [46]. For instance, one may argue against this hierarchical or higher-order view based on experimental demonstrations that the electrical stimulation of the amygdala itself can trigger a subjective experience of fear and anxiety [47]. Although this demonstration was compelling, it is important to mention that in that study this phenomenon occurred only in one out of nine patients. This inconsistency may be partly attributed to inter-individual differences in the spread of electrical activity to other brain regions. However, it is worth noting that the stimulation had a clear dose-dependent effect on the objective physiological response, which was observed across the entire group of patients. Taken together, these results suggest that the amygdala might play a central role in generating physiological responses but possibly a marginal role in generating the conscious experience of fear.

One reason for the skepticism about higher-order models might be that anxiety disorders have been reliably associated with a dysregulation of physiological reactivity [48]. As such, higher-order structures are typically conceptualized as playing more of a complementary role in these pathologies. However, it is worth noting that the therapeutic success of psychotherapies for anxiety and depression appears to be mediated by brain regions such as the dorsomedial prefrontal cortex, posterior cingulate gyrus, precuneus, and some regions of the temporal lobes [49]. Also, recent findings indicated that the inhibition of the amygdala by the dorsolateral prefrontal cortex was positively associated with the outcome of exposure therapy [50]. As such, some higher-order processes may also have an important influence on therapeutic success.

One challenge in the implementation of a higher-order approach to anxiety disorders is the reliance on self-reported measures. This can potentially be a problem, because the means of fear assessment can greatly influence the outcome. We opted for offline categorical ratings, as this is the typical approach for clinical diagnosis, but one may worry that this may not directly reflect the online subjective experience of fear. Our results suggest that both assessment methods may at least partly reflect similar processes, as our decoders trained to predict offline ratings could predict weakly but significantly online ratings in an independent validation fMRI task (Supplementary Methods and Supplementary Fig. S2). However, further work may be needed to determine precisely which aspects of fear are more salient with different means of assessment and how to cover accurately the multiple dimensions relevant to the self-report of fear.

Furthermore, defensive responses are multifaceted and can vary as a function of multiple factors such as the threat imminence. For instance, it was shown that a “reactive network”—primarily including the amygdala, periaqueductal gray, and midcingulate cortex—might be involved in the initial reactivity to threat and rapid escape decisions, whereas a more “cognitive network”—which primarily includes the ventromedial prefrontal cortex, hippocampus, and posterior cingulate cortex—might mainly be involved in generating more complex behavioral responses [51, 52]. Our approach involved relatively simple defensive reactions to visual stimuli and required little interaction with the threat outside of a simple motor response. As such, the cognitive network involved in more complex defensive responses may not have been accurately captured by our analyses.

Another concern is that emotional states have been proposed to involve (and sometimes interfere with) cognitive functions [53]. As such, we can expect part of our results to represent this interaction rather than a strict representation of fear per se. For instance, the middle frontal gyrus has also been involved in the cognitive regulation of emotion [54, 55] and in the regulation of the physiological reactivity network [50]. Furthermore, activity in this region has also been associated with working memory and the retrieval of semantic information [56]. The same logic applies to attentional processes with reported influences in the occipital, frontal, parietal, and ventral temporal regions [57, 58]. As our experiment involved cognitive functions such as working memory and attention, our results may be partly associated with the interference of fear with these cognitive functions [59]. This observation does not undermine our claim, as complex interactions between cognitive and affective processes might also represent an important mechanism of change in psychotherapy [60] that requires further empirical investigation.

Given that multivoxel decoding involves many parameter choices, one may wonder if our results robustly generalize or if they are due to idiosyncratic details. Overall, our impression is the main results do hold up under different analyses (see Fig. 3b, Supplementary Figs. S1 and S2, as well as Supplementary Results). One specific concern is with respect to the choice of a between- or within-subject decoding strategy. Here, both approaches presented some similarities, at least regarding subjective ratings of fear (see Supplementary Methods, Results, and Supplementary Fig. S4). However, the generally weaker performance of within-subject decoders rendered a direct comparison impractical, especially for skin conductance reactivity, which contains too few trials within each participant. Therefore, throughout we primarily focused on the between-subject decoding approach.

Another concern pertains to the use of either binned data or single-trial data. Binned (or averaged) data can be useful to train the decoders as the process of averaging can remove some of the within-subject noise and can make the data manageable for the training procedure. However, it also appears important to test the accuracy of the decoder in the prediction of raw single-trial data. This is why we chose to combine both approaches and to also test our decoders on raw single-trial data (Supplementary Fig. S1).

Another important concern pertains to the generalizability of decoders to other datasets. Our decoders presented good generalization to an independent validation dataset (see Fig. 3b) but also weaker performances on a dataset coming from a different fMRI task (Supplementary Fig. S2). Training the decoders using data from multiple tasks would potentially allow to build decoders that could generalize better across different tasks and datasets.

In sum, we have exploited an opportunity to directly compare how machine-learning decoders can predict the subjective fear rating and its correlated physiological activity. Our results suggest that the study of fear and anxiety disorders may benefit from a greater inclusion of subjective measures, as they might index higher-order processes not readily accessible when studying physiological reactivity alone. This may prove to be an important means to optimize treatments and further tailor interventions to specifically alleviate the subjective suffering associated with fear and anxiety disorders.

## Supplementary information

Supplemental Information

## Data Availability

The data supporting the main findings of this manuscript are available from the ATR repository (https://bicr.atr.jp/decnefpro/) and from the corresponding authors upon reasonable request. Statistical analyses were conducted with Matlab R2017b. fMRI analyses were conducted using SPM 12, pyMVPA 2.4, and the CanLabCore toolbox. Custom code was also used and can be made available upon request.
