# Supplementary material for: Multivoxel pattern analysis reveals dissociations between subjective fear and its physiological correlates
Source: Mol Psychiatry. 2019 Oct 29;25(10):2342–54. doi: 10.1038/s41380-019-0520-3 (PMC7515839; doi:10.1038/s41380-019-0520-3)
Supplement: Supplementary file 1 — Supplemental Information [file 41380_2019_520_MOESM1_ESM.pdf]

Supplemental Information

Multivoxel Pattern Analysis Reveals Dissociations Between Subjective Fear and Its Physiological Correlates

Authors: Vincent Taschereau-Dumouchel [1,2,*] , Mitsuo Kawato [1,3] & Hakwan Lau [1,2,4,5,6,*]

Affiliations:
1- Department of Decoded Neurofeedback, ATR Computational Neuroscience Laboratories, Kyoto, 619-0288, Japan.
2- Department of Psychology, UCLA, Los Angeles, 90095, USA.
3- RIKEN Center for Advanced Intelligence Project, ATR Institute International, Kyoto, Japan.
4- Brain Research Institute, UCLA, Los Angeles, 90095, USA.
5 - Department of Psychology, University of Hong Kong, Pokfulam Road, Hong Kong
6 - State Key Laboratory of Brain and Cognitive Sciences, University of Hong Kong, Hong Kong

* Correspondence to: H.L. (hakwan@gmail.com) and V.T-D.(vincenttd@ucla.edu).

Table of Contents

Supplementary Methods **3**
Apparatus for stimuli presentation 3
Testing whole-brain decoders on single-trial data 3
Testing whole-brain decoders on an independent validation dataset 3
Testing whole-brain decoders on specific phobia patients 4
Assessing the performances of the whole-brain decoders on single-trial images 5
Within-subject decoders 6
The conjunction Analysis 6

Supplementary Results **7**
Testing whole-brain decoders on single-trial data 7
Area Under the Curve 7
Mixed effect models 7
Testing whole-brain decoders on an independent validation dataset 8
Area Under the Curve 8
Mixed effect models 8
Patients presenting specific phobias 9
Within-subject decoders 9
The conjunction Analysis 10

Supplementary Discussion **10**

Supplementary Figures **12**
Figure S1. Whole-brain decoders present sensitive and specific predictions of single-trial data. 12
Figure S2. Whole-brain decoders present weaker, but statistically significant predictions of an independent validation dataset. 13
Figure S3. Decoding accuracy of single-trial images in patients presenting specific phobia. 14
Figure S4. Within-Subject decoding of the subjective fear ratings. 15

Supplementary Table **16**
Table S1. Brain regions consistently involved in the prediction of both the subjective fear ratings and skin conductance responses. 17

Supplementary References **19**

Supplementary Methods

Apparatus for stimuli presentation

Visual stimuli were projected on a translucent screen by an LCD projector (DLA-G150CL, Victor). The projector spanned 20 × 15 deg in visual angle (800 × 600 resolution) and had a refresh rate of 60 Hz. The experiment was conducted using the PsychoPy2 software (v1.83) (Peirce 2007).

Testing whole-brain decoders on single-trial data

To determine the sensitivity of the whole-brain decoders to predict single-trial data, we used a similar leave-one-subject-out decoding approach, but here the test dataset was the single-trial data of the left-out participant. This procedure ensured that the data of the test participants were not included in the training dataset. The single-trial data were selected from the 720 trials that were the first of each series of images of a same category. To allow an unbiased assessment of the performance of the decoders using mixed effect models, the test datasets were balanced to avoid creating skewed distributions. For the subjective fear ratings, we sub-selected, for each participant, an even number of random trials with low (< or equal to ratings of two) and high fear (> or equal to the rating of three). Regarding the skin conductance reactivity, we balanced the dataset using the quintile procedure described above, but applied the decoders to the unaveraged (e.g., single-trial) beta images (i.e., single-trial beta images).

Testing whole-brain decoders on an independent validation dataset

To determine how the whole-brain decoders could generalize to the independent validation data, we tested the prediction of the whole-brain decoders on data from another fMRI experiment. The experiment is described in detail elsewhere (Taschereau-Dumouchel et al. 2018). Briefly, in two fMRI sessions, 17 participants were presented with a total of 40 images of

“feared animals” and 20 images of “non-feared animals” and objects. Offline categorical ratings obtained prior to the experiment were used to determine the animal categories to determine has “fearful” and “non-fearful”. Animal categories associated with “High” or “Very High” fear were selected to be the fearful categories. Following the presentation of each image, fear ratings were collected in the scanner using a 7-point Likert scale. Skin conductance was also recorded during the experiment and the reactivity to each image was established using the same procedure as described in the Methods section.

The preprocessing of the fMRI images was also similar to the preprocessing used in the main experiment: the images were realigned, coregistered, normalized to the MNI space, and smoothed (FWHM = [8,8,8]) using SPM 12. Since this design was not a rapid-event related design (i.e., images were presented for six seconds on the screen), we didn’t use the least-square separate approach and conducted first-level GLMs with one regressor modeling each trial and their time derivative. The resulting single-trial beta images were attributed either their subjective fear rating (i.e., online ratings) or their skin conductance reactivity level. The skin conductance was determined using the procedure described above (i.e., a threshold of 0.2 microsiemens and individual quintiles). Beta images were then standardized and used to test the predictions of the whole-brain decoders.

Testing whole-brain decoders on specific phobia patients

One may expect anxiety disorders to be associated with significantly different brain processes then neurotypical participants (Etkin and Wager 2007). As such, one specific concern is whether the whole-brain decoders could predict accurately the brain data of patients presenting specific phobia. To tackle this question, we recruited three patients (3 females; mean age = 26.33; SD = 7.77) who met the DSM-IV criteria for specific phobia for at least one animal in our database. Patients were diagnosed using the Structured Clinical Interview for DSM-IV

conducted by three medical doctors trained in psychiatric assessment. Diagnoses were established by inter-rater agreement. The patients completed the same fMRI procedure as the neurotypical participants and were presented with 3600 images of animals and objects. As a result, some of the presented images were depicting the feared animal. Following the procedure described above, we obtained single-trial images of the patients. These images were submitted to the whole-brain decoders in order to determine their capacity to predict the subjective fear and skin conductance responses of patients.

Assessing performances of the whole-brain decoders on single-trial images

For each participant and for each decoder, the predictions of the whole-brain decoders were assessed using the area under the ROC curve. This was established in the discrimination of low (< or equal to two) and high (> or equal to three) values of each outcome. The values of the area under the curve were submitted to a repeated-measure ANOVA with two within-subject factors (Decoders and Test dataset) and their interaction. Paired-sample t-tests were also carried out as follow-up analyses.

We also used a second approach to determine if the fear ratings and skin conductance reactivity (i.e., real values) could be accurately predicted by the decoders. For each decoder, we carried out a two-level mixed effect model predicting the real values of the outcome using three fixed effects (predicted values of the decoder, test dataset, predicted values X test datasets). We also allowed the intercepts to have a random component in order to correct for potential clustering of error within-participants (Raudenbush and Bryk 2002). The predicted values of the decoders were standardized within participant. This approach allowed to first test for an interaction between the predicted values and the test dataset in the prediction of the real values. Follow-up analyses were carried using two-level mixed effect models predicting the real values

using one fixed effect (predicted values) and one random component (the within-participant intercept).

Within-subject decoders

We also trained within-subject decoders to determine if the fine-grained brain representations also followed a similar pattern as the one observed using the between-subject decoders. This, however, poses a challenge regarding the skin conductance reactivity decoder because a much smaller volume of data is available since skin conductance responses occurred on average on 28.21% of trials. As a result, we present here only the within-subject decoders of the subjective fear ratings and tried to determine if a similar pattern of accuracy could be observed as with the between-subject decoders. Accordingly, we conducted within-subject decoding in the significant regions previously reported in Figure 4b and tried to determine if similar results could be observed within-subject (i.e. better decoding of subjective fear ratings in the significant regions of the middle frontal gyrus than the significant regions of the amygdala, insula and ventral middle prefrontal cortex).

The data used to train within-subject decoders were preprocessed in the same way as the data used to train the between-subject decoders, but were not smoothed. Within-subject data were binned as a function of block and subjective fear ratings. We used support vector regression and a leave-one-block-out cross-validation approach.

The conjunction Analysis

We aimed to determine if some brain regions could accurately predict both subjective fear and skin conductance reactivity. Using the within-region decoding procedure, we established the brain regions significantly associated with the prediction of both outcomes. The significance was established by extending the permutation approach used in the within-region

decoding to obtain a distribution of maximum AUCs under the null hypothesis. As such, the labels of the data were permuted 1,000 times and the AUC associated with each random permutation was determined. The distribution of maximum AUCs was constructed by selecting, for each iteration, the maximum AUC value across all 214 ROIs (i.e., 1,000 maximum values). The obtained critical values (*P* = .05) was determined both for the subjective fear decoders (critical AUC = .658) and the skin conductance decoders (critical AUC = .666). The critical values were used to determine the significant regions for each outcome. The regions presenting a significant prediction of both outcomes are reported in Table S1.

Furthermore, we aimed to determine if the brain representations were also similar within each significant region. This was achieved by using the cross-decoding procedure which involved testing each decoder using the dataset of the other outcome (e.g., testing the subjective fear rating decoder using the skin conductance dataset). Using the same permutation procedure as described above, we determined critical AUC values for the decoding of subjective fear data using the skin conductance decoders (AUC = .657) and for the decoding of skin conductance data using the subjective fear decoders (AUC = .663). We established if any regions presenting a significant prediction of both outcomes also presented significant cross-decoding.

Supplementary Results

Testing whole-brain decoders on single-trial data

Area Under the Curve

The results of the repeated-measure ANOVA showed a significant decoder X test dataset interaction ($F(1,24) = 62.17$; $P < .0001$; two-sided) (see Figure S1). Each decoder presented greater areas under the curve when tested on their corresponding dataset (Subjective fear decoder: $t(24) = 4.68$; $P < .0001$; two-sided; Skin conductance reactivity

decoder: t(24) = -7.55; P < .0001; two-sided) (see Figure S1 a and b). The fear decoder presented greater AUCs when tested on the fear dataset (M = .60; SD = .055) then on the skin conductance reactivity dataset M = .53; SD = .070). Similarly, the skin conductance reactivity decoder presented greater AUCs when tested on the skin conductance reactivity dataset (M = .62; SD = .050) then on the fear dataset (M = .52; SD = .043). The effect sizes were considered to be of large sizes both for the subjective fear decoder (Cohen's d = 0.94) and for the skin conductance reactivity decoder (Cohen's d = 1.51) (corrected for dependence between the means) (Morris and DeShon 2002). The data met the assumptions of the test. No outlier was observed and the data was normally distributed.

Mixed effect models

Likewise, we observed a significant interaction between the predicted values and the test datasets both for the subjective fear (predicted values X test datasets interaction: $t(16757) = -8.01$; $P < .0001$; two-sided) and the skin conductance reactivity decoders (predicted values X test datasets interaction: $t(14720) = -14.12$; $P < .0001$; two-sided). These results indicate that both decoders present a better prediction of their corresponding dataset at the single-trial level. More precisely, the subjective fear decoder predicts more accurately the real values of the subjective fear dataset ($t(11681) = 23.77$; $P < .0001$; two-sided) than the skin conductance dataset ($t(5076) = 5.01$; $P < .0001$; two-sided). Similarly, the skin conductance reactivity decoder predicts more accurately the real values of the skin conductance reactivity dataset ($t(5076) = 20.17$; $P < .0001$; two-sided) than the subjective fear dataset ($t(9644) = 4.46$; $P < .0001$; two-sided).

The data generally met the assumptions of the test, which were assessed within each participant (Tabachnick and Fidell 2012). No outlier was observed on the dependent variable. Some participants presented outliers on the predicted values (> |3| SD) but these were mainly

expected given the number of observations. The data is also generally normally distributed with 2 participants presenting moderately skewed distribution (+/- 0.5 to 1.0; both for the predicted values of the SCR and Fear decoders) and 3 participants presenting a moderately leptokurtic distribution (> |1|; both for the predicted values of the SCR and Fear decoders). There was no multicollinearity, and no participants presented clear sign of heteroscedasticity. Furthermore, for all participants, the residual error appeared to be independent from the case order. There was also a few outliers (> |3| SD) in the residuals (> |3| SD: SCR = 7; Fear = 11).

Testing whole-brain decoders on an independent validation dataset

Area Under the Curve

The results of the repeated-measure ANOVA showed no significant decoder X test dataset interaction ($F(1,16) = .669$; $P = .426$; two-sided). There was also no statistically significant difference between the test datasets for each decoder (Subjective fear decoder: $t(17) = .775$; $P = .450$; two-sided; Skin conductance reactivity decoder: $t(17) = -612$; $P = .549$; two-sided). The mean values were although qualitatively in the same direction as the ones obtained with the single-trial data (see Figure S2). The data met the assumptions of the test. No outlier was observed and the data was normally distributed.

Mixed effect models

Likewise, we observed no interaction between the predicted values and the test datasets both for the subjective fear (predicted values X test datasets interaction: $t(1306) = -1.03$; $P = .30$) and the skin conductance reactivity decoders (predicted values X test datasets interaction: $t(1306) = -1.59$; $P = 0.11$; two-sided). However, both decoders present a statistically significant prediction of their corresponding dataset. More specifically, the subjective fear decoder presented a statistically significant prediction of the subjective fear dataset ($t(803) = 2.87$; $P = .004$; two-sided) and not of the skin conductance dataset ($t(503) = 1.35$; $P = .17$; two-sided).

Similarly, the skin conductance reactivity decoder present a statistically significant prediction of the real values of the skin conductance dataset ($t(503) = 2.38$; $P = .018$; two-sided) and not of the subjective fear dataset ($t(805) = -0.019$; $P = .98$; two-sided) (see Figure S3 c and d).

The data generally met the assumptions of the test, which were assessed within each participant (Tabachnick and Fidell 2012). No outlier was observed on the dependent variable. 2 participants presented outliers on the predicted values (> |3| SD; both for the predicted values of the SCR and Fear decoders). The data is also generally normally distributed: only a few participants (2 participants for the Fear decoders and 3 for the SCR decoder) presented moderately skewed distribution (+/- 0.5 to 1.0) and no participants presented kurtosis values deviating from the expected values. There was no multicollinearity and no participants presented a clear sign of heteroscedasticity. Furthermore, for all participants, the residual error appeared to be independent from the case order. Also, there was no outlying values in the residuals (> |3| SD).

Patients presenting specific phobias

Whole-brain decoders were used to predict the single-trial images of the patients. The areas under the curve of the predicted values are presented in Figure S3. To quantify the divergence of patients with respect to the group of participants, we used the standard deviation of the distributions. For the subjective fear decoder, the areas under the curve were included within 0.53 and -0.5 standard deviations for both test datasets. With respect to the skin conductance reactivity decoder, the areas under the curve of patients are included within 1.85 and -0.56 standard deviations for both test dataset. While this is a restricted sample, these results suggest that whole-brain decoders do not perform significantly worse when predicting data of patients. The results even suggest that the skin conductance reactivity of some patients might be predicted more efficiently than the rest of the group (z scores of 1.38 and 1.85).

Within-subject decoders

Within-subject decoders were trained to predict the subjective fear ratings in the brain regions previously reported as significant in Figure 4b. The results are presented in Figure S4. A similar tendency as with between-subject decoders can be observed: the significant regions of the middle frontal gyrus tend to present a greater accuracy in the prediction of the fear ratings than the other significant regions of the amygdala, insula and ventromedial prefrontal cortex ($t(30) = 1.79$; $P = .08$; two-sided). It is also to be noted that the within-subject decoders were significantly less accurate than the between-subject decoders ($t(8) = 2.35$; $P = 0.046$; two-sided). This is to be expected as within-subject decoders are trained with a subsample of the data. The data met the assumptions of the tests. No outlier was observed and the data was normally distributed.

The conjunction Analysis

Table S1 indicates the brain regions in which the predictions of both outcomes were statistically significant. These regions were located in the inferior frontal gyrus (inferior frontal sulcus), the superior temporal gyrus (TE1.0, TE1.2, and rostral area 22), the fusiform gyrus (rostroventral area 20 and lateroventral area 37), the parahippocampal gyrus (area TL), the posterior superior temporal sulcus (caudoposterior superior temporal sulcus), the superior parietal lobule (rostral area 7), the precuneus (median area 5 and area 31), the insular gyrus (dorsal granular insula), the medioventral occipital gyrus (caudal lingual gyrus, rostral cuneus gyrus, and ventromedial parietooccipital sulcus), and the right hippocampus (rostral and caudal hippocampus). None of these significant regions also presented a significant cross-decoding for both decoders. However, two regions – the rostral area 22 of the superior temporal gyrus and the area TL of the parahippocampal gyrus – presented results that were close to significance.

Supplementary Discussion

The supplementary analyses provided information regarding the accuracy of whole-brain decoders to predict the single-trial images both of the discovery cohort and the independent validation. The results indicate that whole-brain decoders present sensitive and specific predictions of the single-trial data of the discovery cohort. Also, when whole-brain decoders are applied to the independent validation data, their predictions are weaker, but both decoders still present a statistically significant prediction of their corresponding dataset. This weaker performance is to be expected since the independent validation dataset was acquired using a different fMRI task that potentially engaged different brain processes. As such, during this experiment, participants were not required to perform a 1-back task on the image category and were explicitly asked to rate their subjective fear of the presented images. Also, the independent validation dataset was substantially smaller than the original dataset which likely decreased the statistical power of these tests.

The supplementary analyses also suggested that the whole-brain decoders might present similar levels of accuracy in patients presenting specific phobia. More precisely, no patients presented prediction accuracies significantly below the group mean (all above -.53 standard deviation). While these results were obtained with a small number of patients (N = 3) they still provide some evidence suggesting that the whole-brain decoders of subjective fear ratings and skin conductance reactivity might generalize to patients. Although, further analyses will be necessary to assess this claim with greater statistical rigor.

Also, the supplementary analyses indicate a tendency for a better decoding of subjective fear ratings from the middle prefrontal cortex as opposed to the amygdala, insula, and ventromedial prefrontal. However, the interpretation of this marginally significant result is limited by the relative weakness of the decoding performance in the within-subject analyses. This weak

decoding performance and the small number of skin conductance responses obtained within-subjects prevented further investigation of the difference in the accuracies of within-subject decoders. As a result, further investigations will be needed to provide more conclusive evidence regarding the role of the fine-grained information in the decoding of subjective fear ratings and skin conductance reactivity.

Lastly, the results of the exploratory conjunction analysis revealed that some brain regions were associated with the prediction of both the subjective fear ratings and skin conductance reactivity. These regions notably include part of the inferior frontal gyrus, insula (i.e., dorsal granular insula), precuneus, hippocampus, and fusiform gyrus. These results suggest the interesting possibility that similar brain processes might be recruited by both conditions. At least three different functions are likely to be involved. First, both physiological reactivity and subjective fear are likely to increase selective attention toward visual stimuli. As such, the conjunction of significant predictions in early visual areas and the ventral temporal cortex might partly reflect this well-documented attentional effect (Vuilleumier et al. 2001; Armony and Dolan 2002). Second, both outcomes are also susceptible to recruit brain regions involved in the processing of salient stimuli. This possibility is notably supported by the involvement of the insula, which has been proposed as one of the key nodes of the salience network (Uddin 2015). Third, it is also possible that both subjective fear and physiological reactivity might have recruited similar regulatory processes. For instance, it has been shown that brain regions such as the inferior frontal gyrus (Braunstein, Gross, and Ochsner 2017; Buhle et al. 2014) might be involved in both the implicit and explicit regulation of emotional processes.

However, it is also important to mention that these significant regions might carry very different processes in both conditions. Importantly, the results of the cross-decoding analysis indicated that none of the significant regions also presented a significant cross-decoding for

both decoders. Only two brain regions – the rostral area 22 of the superior temporal gyrus and the area TL of the parahippocampal gyrus – presented results that were close to statistical significance.

In sum, the results of this conjunction analysis suggest the interesting possibility that some brain processes might be shared between the two outcomes. However, our experiment was not designed to offer very detailed information on the nature of these common processes and further experiments will be required to offer a more thorough answer.

Supplementary Figures

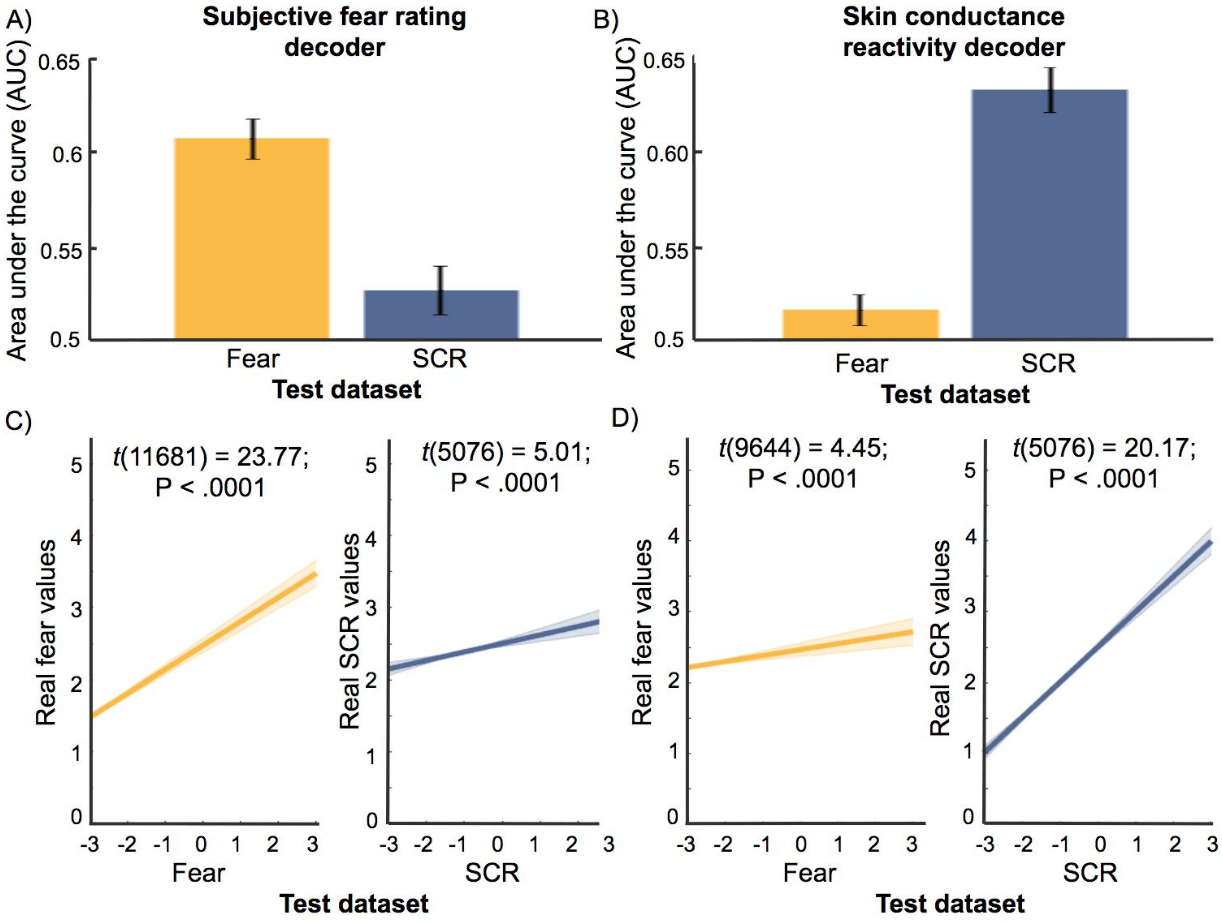

Figure S1. Whole-brain decoders present sensitive and specific predictions of single-trial data.

Whole-brain decoders of the subjective fear rating (left panels) and the skin conductance reactivity (right panels) present more accurate predictions when tested on the dataset they were trained to predict (e.g., subjective fear decoder predicting fear data). This is illustrated both by the use of the areas under the curve (top panels) and by mixed-effect models (bottom panels). Error bars are +/- 1 S.E.M. Shaded error bars correspond to the 95% confidence intervals of the slope and intercept of the mixed effect models.

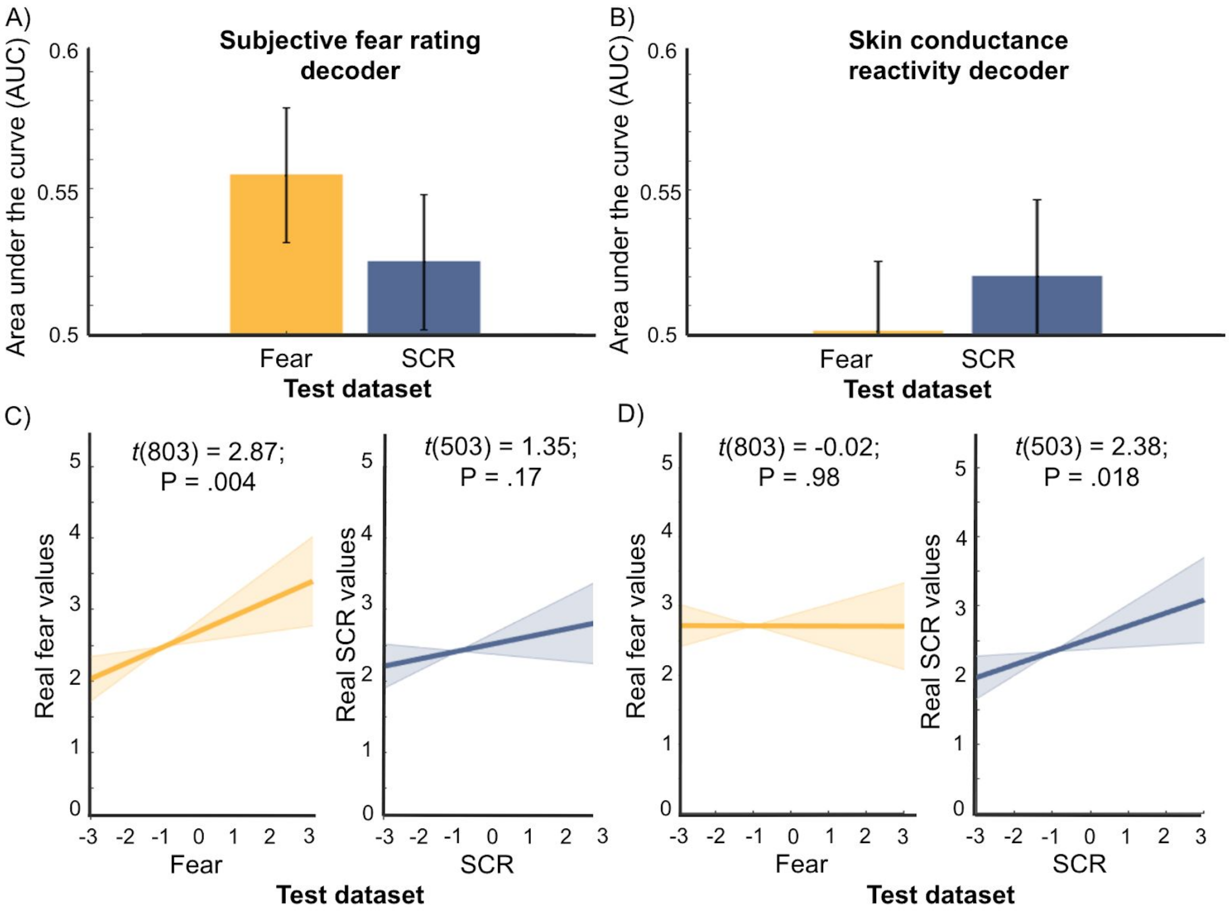

Figure S2. Whole-brain decoders present weaker, but statistically significant predictions of an independent validation dataset.

Using the area under the ROC curve (a and b), the whole-brain decoders of the subjective fear rating (left panels) and the skin conductance reactivity right panels) do not present a significant prediction of their corresponding dataset. Although, mixed-effect models (c and d) indicate a weak, but statistically significant capacity of the decoders to predict their corresponding datasets. These results are qualitatively in line with the single-trial results of the discovery cohort (i.e., greater capacity to predict the corresponding dataset). Error bars are +/- 1 S.E.M. Shaded error bars correspond to the 95% confidence intervals of the slope and intercept of the mixed effect models.

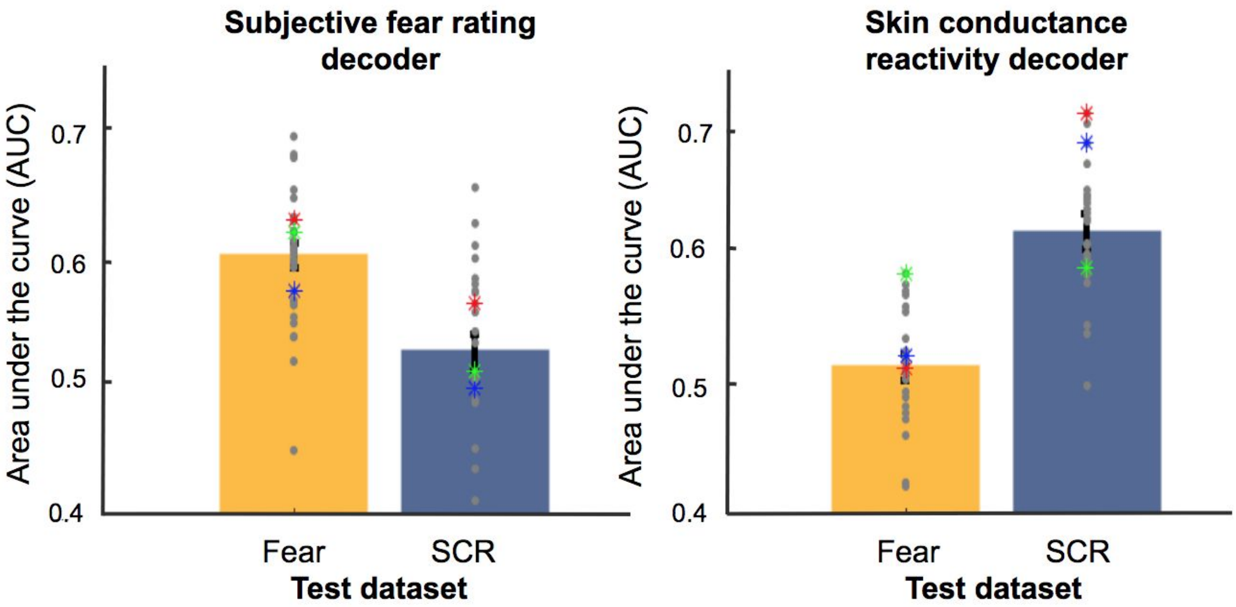

Figure S3. Decoding accuracy of single-trial images in patients presenting specific phobia.

Colored stars represent the area under the curve of patients presenting specific phobias. No patients presented areas under the curve below 0.56 standard deviation. As such, these results do not indicate a clear decrease in the capacity to predict the single-trial values of patients. Error bars are +/- 1 S.E.M.

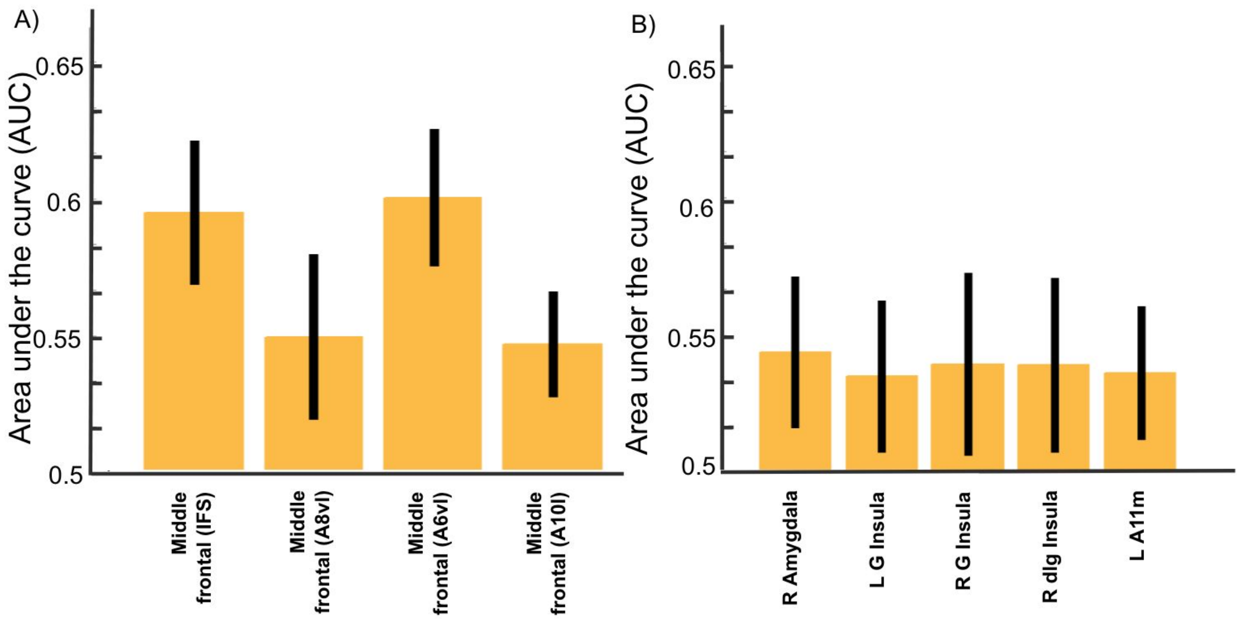

Figure S4. Within-Subject decoding of the subjective fear ratings.

Within-subject decoders were less accurate than the between subject decoders in the prediction of the subjective fear ratings ($t(8) = 2.35$; $P = 0.046$; two-sided). However, they still show a similar tendency to present better performances in the middle frontal gyrus than the amygdala, insula, and ventromedial prefrontal cortex ($t(30) = 1.79$; $P = .08$; two-sided). Error bars are +/- 1 S.E.M.

Supplementary Table

Gyrus	Region	Laterality	MNI coordinates [x, y, z]	Mean AUC	Fear decoder (AUC)	SCR decoder (AUC)
Inferior frontal	inferior frontal sulcus	L	[-47, 32, 14]	0.699	0.717	0.681
Superior temporal	TE1.0 and TE1.2	R	[51, -4, -1]	0.666	0.697	0.737
	A22r, rostral area 22	L	[-55, -3, -10]	0.701	0.658	0.743
Fusiform	A20rv, rostroventral area 20	R	[33, -15, -34]	0.734	0.703	0.764
	A37lv, lateroventral area37	R	[43, -49, -19]	0.705	0.664	0746
Parahippo-campal	TL, area TL	L	[-28, -32, -18]	0.705	0.722	0.688
posterior Superior Temporal Sulcus	cpSTS, caudoposterior superior temporal sulcus	L	[-52, -50, 11]	0.733	0.774	0.693
Superior parietal lobule	A7r, rostral area 7	L	[-16, -60, 63]	0.692	0.698	0.685
Precuneus	A5m, medial area 5(PEm)	L	[-8, -47, 57]	0.688	0.669	0.708
		R	[7, -47, 58]	0.710	0.696	0.724
	A31, area 31 (Lc1)	L	[-6, -55, 34]	0.675	0.683	0.668
Insular	dIg, dorsal granular insula	L	[-38, -8, 8]	0.736	0.712	0.761
MedioVentral Occipital	cLinG, caudal lingual gyrus	L	[-11, -82, -11]	0.740	0.744	0.736
		R	[10, -85, -9]	0.714	0.725	0.702
	rCunG, rostral cuneus gyrus	L	[-5, -81, 10]	0.726	0.677	0.775

	vmPOS,ventromedial parietooccipital sulcus	L	[-13, -68, 12]	0.738	0.816	0.675
Hippocampus	rostral and caudal hippocampus	R	[22, -12, -20]<br>[29, -27, -10]	0.746	0.816	0.675

Table S1. Brain regions consistently involved in the prediction of both the subjective fear ratings and skin conductance responses.

Supplementary References

Etkin, Amit, and Tor D. Wager. 2007. “Functional Neuroimaging of Anxiety: A Meta-Analysis of Emotional Processing in PTSD, Social Anxiety Disorder, and Specific Phobia.” *The American Journal of Psychiatry* 164 (10): 1476–88.

Morris, Scott B., and Richard P. DeShon. 2002. “Combining Effect Size Estimates in Meta-Analysis with Repeated Measures and Independent-Groups Designs.” *Psychological Methods* 7 (1): 105–25.

Peirce, Jonathan W. 2007. “PsychoPy--Psychophysics Software in Python.” *Journal of Neuroscience Methods* 162 (1-2): 8–13.

Raudenbush, Stephen W., and Anthony S. Bryk. 2002. *Hierarchical Linear Models: Applications and Data Analysis Methods*. SAGE.

Tabachnick, Barbara G., and Linda S. Fidell. 2012. *Using Multivariate Statistics*. Prentice Hall.

Taschereau-Dumouchel, Vincent, Aurelio Cortese, Toshinori Chiba, J. D. Knotts, Mitsuo Kawato, and Hakwan Lau. 2018. “Towards an Unconscious Neural Reinforcement Intervention for Common Fears.” *Proceedings of the National Academy of Sciences of the United States of America* 115 (13): 3470–75.
